# Description and biomechanical evaluation of the modified laparoscopic-assisted percutaneous gastropexy technique in dogs

**DOI:** 10.3389/fvets.2024.1509728

**Published:** 2025-02-03

**Authors:** Dong Woo Kim, Ho Hyun Kwak, Junhyung Kim, Heung Myong Woo

**Affiliations:** ^1^Department of Veterinary Surgery, College of Veterinary Medicine, Institute of Veterinary Science, Kangwon National University, Chuncheon, Republic of Korea; ^2^Department of Companion Animal Industry, College of Natural and Life Sciences, Daegu University, Gyeongsan, Republic of Korea

**Keywords:** laparoscopy, minimally invasive surgery, prophylactic gastropexy, gastric dilatation and volvulus, laparoscopic gastropexy, barbed suture

## Abstract

**Background:**

Total laparoscopic gastropexy (TLG) has become increasingly popular due to its minimally invasive nature, requiring only three ports and no additional skin incisions aside from those for port placement. However, a notable limitation of TLG is the difficulty and time required for intracorporeal suturing. To address this challenge, we investigated a new technique—modified laparoscopic-assisted percutaneous gastropexy (mLAPG)—in canine cadavers.

**Materials & methods:**

Twelve canine cadavers were divided into mLAPG (*n* = 6) and TLG (*n* = 6) groups. mLAPG was performed using a knotless barbed suture with two ports, and TLG was performed using a single-layer continuous barbed suture. Both methods employed a suture length of 3 cm and four suture bites. The total surgical time (TST) and gastropexy suturing time (GST) were recorded. Upon completion of the procedure, the stomach and body wall, including pexy site, were collected to evaluate the maximum load to failure of the gastropexies.

**Results:**

No significant differences were observed in the TST between the mLAPG (61.83 ± 4.80 min) and TLG (65.33 ± 12.05 min) groups (*p* = 0.538). The GST showed no significant difference between the mLAPG group (31.33 ± 3.13 min) and the TLG group (37.5 ± 7.06 min) (*p* = 0.095). The mLAPG group (35.86 ± 8.24 N) had a significantly higher maximum load to failure than the TLG group (24.04 ± 7.16 N) (*p* = 0.024).

**Conclusion:**

The results of this study suggest that the mLAPG, with its minimal invasiveness, absence of an intracorporeal suturing process, and high tensile strength can be clinically applied for gastropexy in dogs. However, further clinical trials are warranted to further validate this technique and confirm its effectiveness.

## 1 Introduction

Gastric dilatation and volvulus (GDV) is a lethal condition that mostly occurs in large, deep-chested dogs (1). Even if prompt diagnosis and immediate surgical intervention is completed, GDV has devastating consequences. However, GDV can be prevented by prophylactic gastropexy (2, 3). Prophylactic gastropexy seeks to establish a lasting connection between the pyloric antrum and right internal abdomen; numerous methods have been developed to achieve this (2, 4–14). Several minimally invasive approaches currently exist, including laparoscopic-assisted gastropexy (12) and total laparoscopic gastropexy (TLG) (2, 3, 5, 6, 8, 11, 15). TLG is gaining popularity owing to its low morbidity, rapid recovery, and successful adhesion (2, 8, 15). However, laparoscopic intracorporeal suturing is a challenging and time-consuming aspect of this technique (9, 16).

The percutaneous internal ring suturing (PIRS) technique uses laparoscopic-assisted percutaneous knotting and was first described in 2006 for repairing inguinal hernias in children (17, 18). The PIRS is an endoscopic technique that involves closing the internal ring percutaneously with a suture, under the control of a laparoscope. Various laparoscopic techniques have been developed to repair inguinal hernias in human medicine (19).

However, since its introduction, PIRS has become widely used for inguinal hernia surgery because it is relatively easy to learn, reduces operation time, and comparable risk of complications or recurrences compared to intracorporeal suturing. Additionally, it provides an excellent cosmetic outcome by utilizing a single-port approach (20, 21). Owing to these benefits, this technique has been applied to anterior gastropexy to treat both acute and chronic gastric volvulus in infants by adding one instrumental port to manipulate the stomach and suture materials (22).

In veterinary medicine, several studies have utilized the PIRS (21, 23, 24). The anterior gastropexy technique, initially adapted from PIRS for use in infants, has been further modified and applied to dogs. In modified laparoscopic-assisted percutaneous gastropexy (mLAPG), a barbed suture is used to induce permanent adhesion without incision of the pexy site and higher load to failure.

This study aimed to assess the technical feasibility of mLAPG by examining the total surgical time (TST) and gastropexy suturing time (GST), as well as evaluating the biomechanical properties of the pexy site by measuring the maximum load to failure. The outcomes were then compared to those of TLG using a canine cadaver model in an acute setting. To the best of our knowledge, this is the first study describing a novel two-port mLAPG technique in dogs.

## 2 Materials and methods

### 2.1 Specimen preparation

All procedures in the study were approved by the Institutional Animal Care and Use Committee of Kangwon National University (IACUC number: KW-231226-2). Twelve freshly thawed canine cadavers that were euthanized for reasons unrelated to the present study were included. The cadavers were randomized into the mLAPG (*n* = 6) and TLG (*n* = 6) groups. All surgical procedures were performed by the same person (D-WK). The dogs were positioned in dorsal recumbency on the surgical table and the ventral abdominal area was clipped and prepared. Using the Veress needle technique, the abdomen was insufflated with carbon dioxide (CO_2_) at a pressure of 10–12 mmHg (Endo Arthroflator VET, Karl Storz Veterinary Endoscopy, Tuttlingen, Germany).

### 2.2 Total laparoscopic gastropexy

Three ports were placed at the ventral midline. After the transabdominal stay suture, the seromuscular layer of the stomach and abdominal wall muscle were sutured in accordance with the manufacturer’s instructions using an absorbable knotless unidirectional barbed suture (2-0 V-Loc 180, 45 cm barbed suture, Covidien, Dublin, Ireland) in a simple continuous pattern with four suture bites and a 3-cm suture length. After deflation of the abdomen, the stay suture was removed, and the port sites were closed in a standard manner.

### 2.3 Modified laparoscopic assisted percutaneous gastropexy

**Figure 1:**
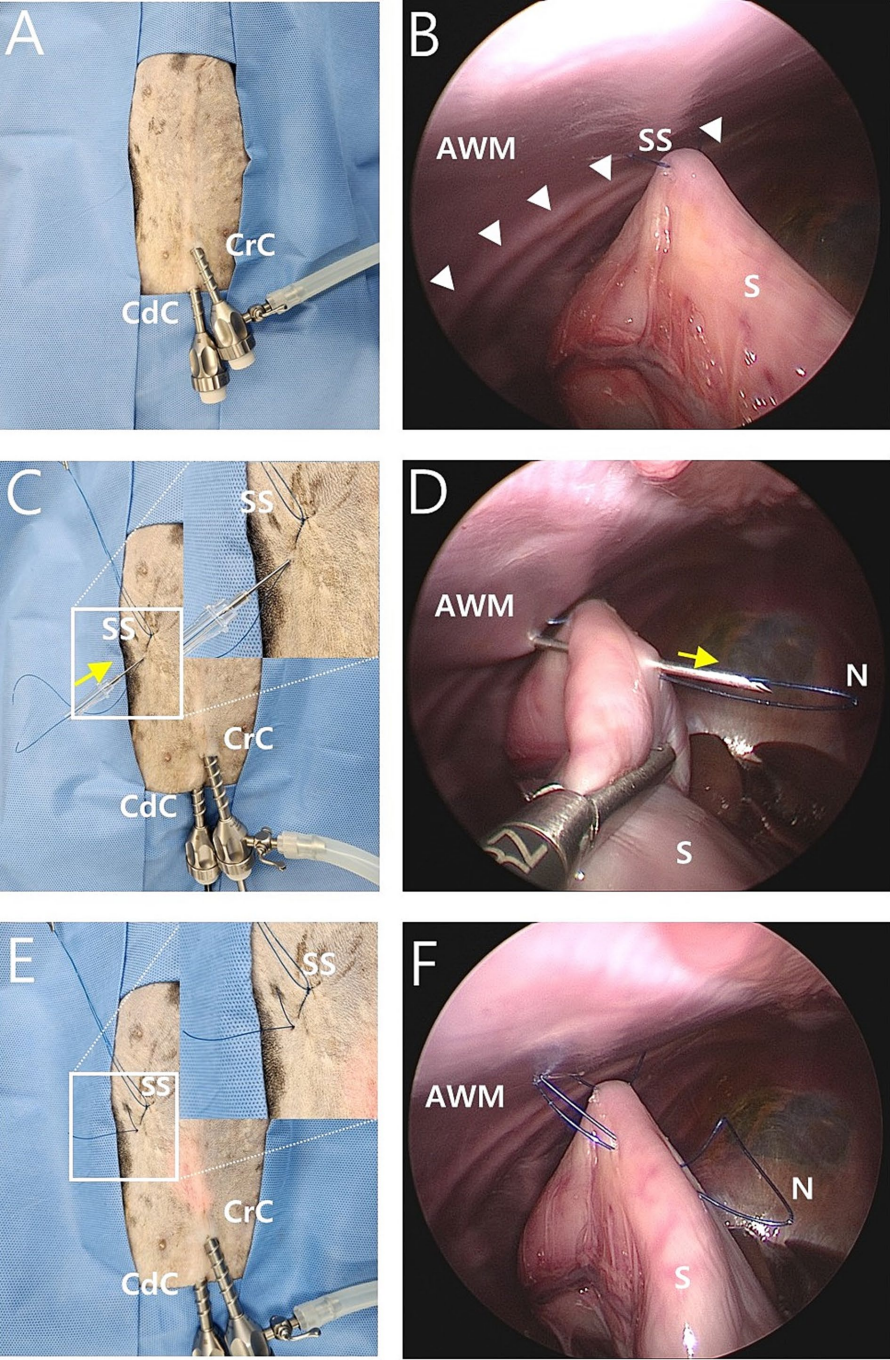
Intraoperative image of modified laparoscopic assisted percutaneous gastropexy (mLAPG) in a canine cadaver. **(A)** An external image of laparoscopic portal placement. The cranial cannula was inserted immediately caudal to the umbilicus and a caudal cannula was inserted 3 cm caudal to the cranial cannula. **(B)** A laparoscopic image of the transabdominal stay suture positioned 2 cm caudal 13th rib (arrow heads) and 5 cm to the right of the ventral midline. The pyloric antrum is suspended against the abdominal wall. External images of the stay suture (SS) are shown in **C** and **E**. **(C,D)** The needle of a 16-gauge intravenous catheter (direction is marked by the yellow arrow) with a 1-0 nylon loop (N) through it was introduced through the abdominal wall into the abdominal cavity. With the movement of the needle of the 16-gauge intravenous catheter and laparoscopic grasping forceps, the needle of the 16-gauge intravenous catheter passed through the seromuscular layer of the stomach. **(E,F)** The needle of a 16-gauge intravenous catheter was removed out of the abdominal cavity, leaving the nylon loop (N) inside the abdominal cavity. The external portions of the nylon loop (N) are shown. CrC, cranial cannula; CdC, caudal cannula; AWM, abdominal wall muscle; S, stomach; SS, stay suture; N, nylon loop.

**Figure 2:**
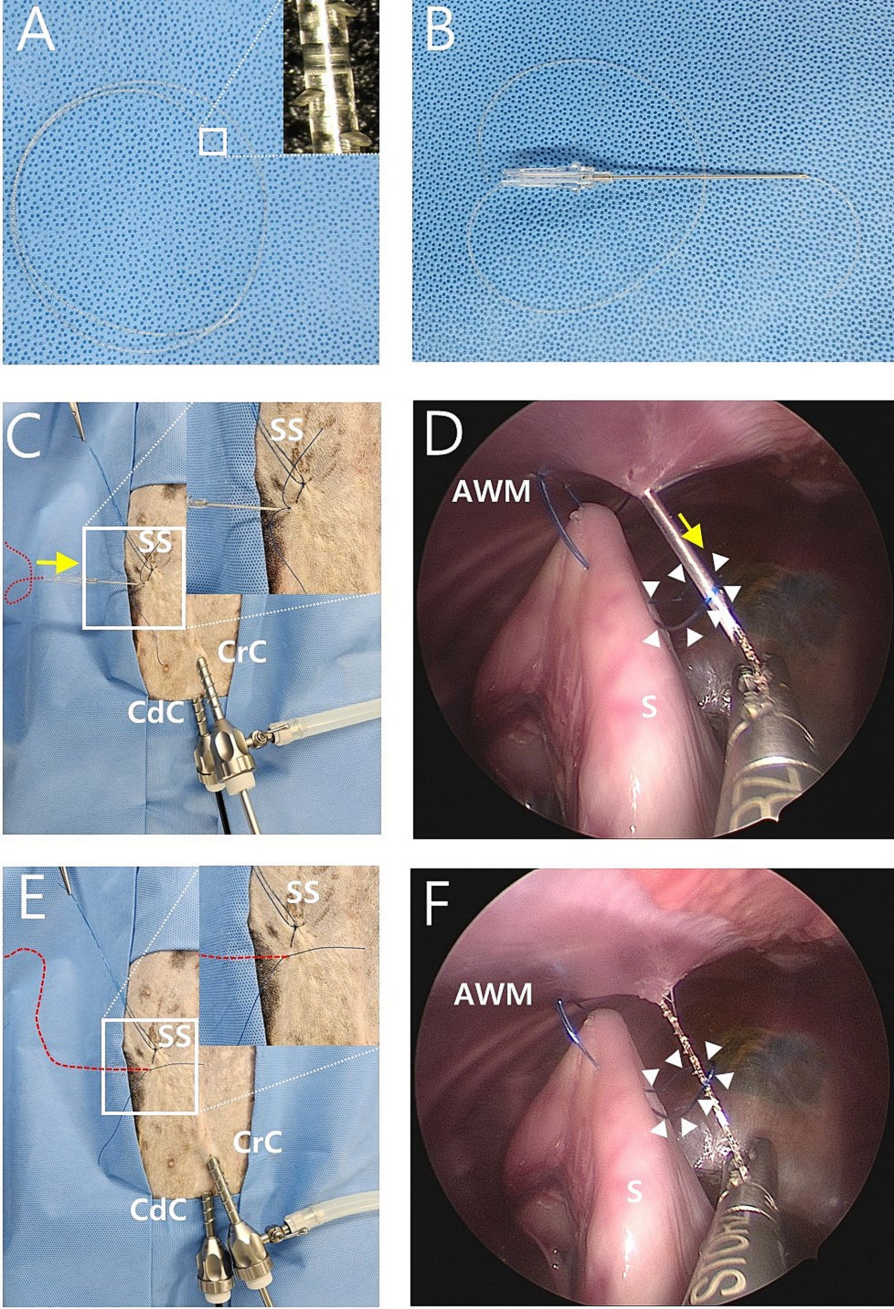
Intraoperative image of modified laparoscopic assisted percutaneous gastropexy (mLAPG) in a canine cadaver. **(A)** A 2-0 knotless barbed suture of which the needle and loop was removed. The magnified image of the suture is shown. **(B)** The needle of the 16-gauge intravenous catheter through which the 2-0 barbed suture was passed into the barrel of the needle. **(C,D)** The needle of a 16-gauge intravenous catheter with a barbed suture (red dotted line) through it introduced into the skin puncture point where the needle previously inserted and into the nylon loop (arrow heads) previously formed in the abdomen. The 2-0 barbed suture is marked with a red dotted line due to its undyed color. The direction is marked by the yellow arrow. **(E,F)** The 16-gauge needle was withdrawn while the end of the 2-0 barbed suture (red dotted line) passing through the inside of the nylon loop (arrow heads) is grasped by laparoscopic forceps. CrC, cranial cannula; CdC, caudal cannula; AWM, abdominal wall muscle; S, stomach; SS, stay suture.

We used an mLAPG technique which involved modifying the suture material, suture length, number of sutures, and pexy site to apply laparoscopic-assisted percutaneous anterior gastropexy technology in infants (22) to gastropexy in dogs. The two-port technique was performed at the caudal midline of the abdomen. The first 6 mm cannula (Ternamian Endotip Cannula, Karl Storz Veterinary Endoscopy, Tuttlingen, Germany) was inserted just caudal to the umbilicus using a blind technique. A second 6 mm cannula (Ternamian Endotip Cannula) was inserted 3–5 cm caudal to the umbilicus (Figure 1A). Then, a 5 mm HOPKINS^®^ II Straight Forward Telescope 0° (Karl Storz Veterinary Endoscopy, Tuttlingen, Germany) was introduced into the cranial cannula and 5 mm endoscopic fundus grasping forceps (33821FG, Karl Storz Veterinary Endoscopy, Tuttlingen, Germany) were inserted in the caudal cannula. An avascular region of the pyloric antrum was grasped with the fundus-grasping forceps midway between the greater and lesser curvatures and positioned approximately 2 cm behind the 13th rib and 5 cm to the right of the ventral midline, where there was not much tension. Then, a 1-0 nylon suture was used to create a transabdominal stay suture (Figure 1B) by making a plica of the stomach, checking for mucosal slip, and passing the nylon suture through the seromuscular layer with caution. The thread of the knotless barbed suture was prepared by removing the needle and loop of the suture (Figure 2A).

The location for the needle puncture was selected from the camera view by pressing the skin from the outside. Then, the needle of a 16-gauge intravenous catheter (BD Angiocath Plus; Becton-Dickinson and Company, Franklin Lakes, NJ) with a 1-0 nylon thread loop was introduced through the abdominal wall into the abdominal cavity (Figure 1C). With movement of the needle and laparoscopic fundus-grasping forceps, the tip of the needle passed through the seromuscular layer of the stomach under a laparoscopic camera view (Figure 1D). To avoid penetrating the lumen of the stomach, the seromuscular layer was grasped with laparoscopic fundus grasping forceps, and the felt mucosa slipped down. The needle was removed from the abdominal wall (Figure 1E), leaving a 1-0 nylon loop inside the abdomen (Figure 1F).

**Figure 3:**
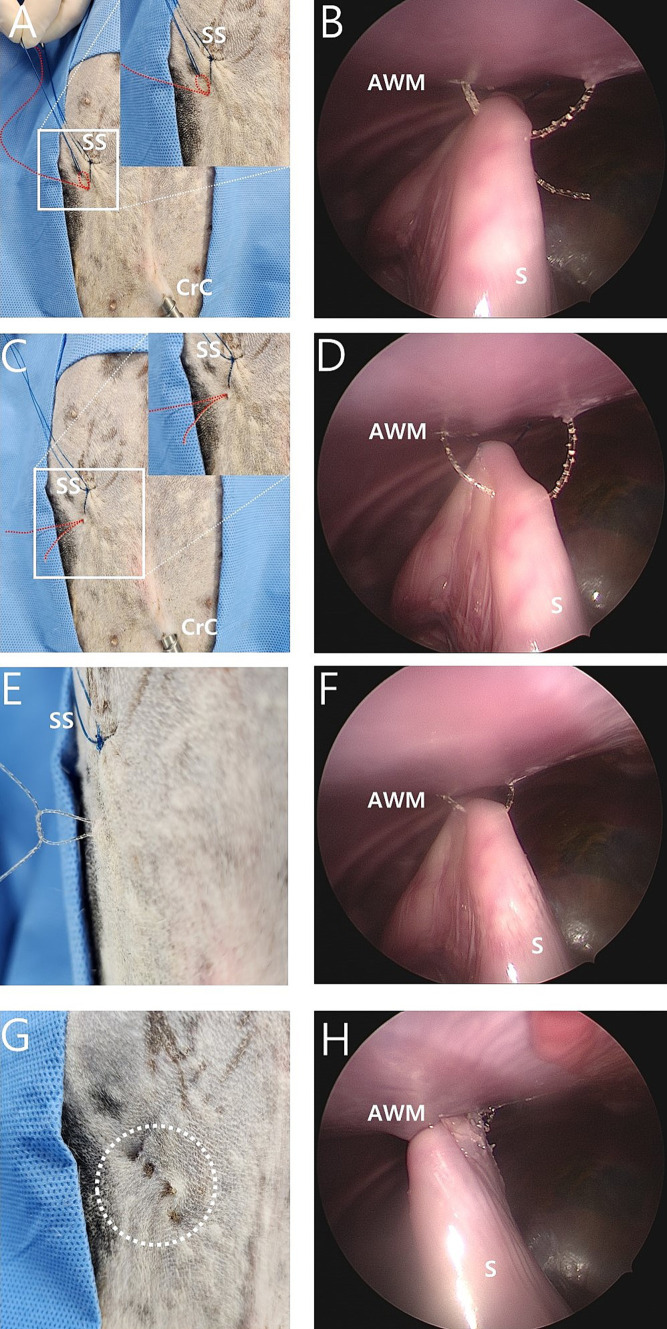
Intraoperative image of modified laparoscopic assisted percutaneous gastropexy (mLAPG) in a canine cadaver. **(A,B)** The process of manually pulling externally the nylon loop made the 2-0 barbed suture (red dotted line) attracted externally through the path which previously passed by the nylon thread. **(C,D)** The nylon was completely pulled out of the body cavity, with the 2-0 barbed suture (red dotted line) passing through the seromuscular layer of the stomach. Both ends of the 2-0 barbed suture (red dotted line) emerged through the same skin point. **(E,F)** The 2-0 barbed suture emerged from the skin and was tied with a square knot. **(G,H)** The white dotted circle indicates the surgical site with a total of four sutures completed. The distance from the most cranial knot to the most caudal knot is 3 cm. CrC, cranial cannula; CdC, caudal cannula; AWM, abdominal wall muscle; S, stomach; SS, stay suture.

The needle of the 16-gauge intravenous catheter with the barbed suture was passed through the same skin puncture point as previously used (Figures 2B,C). The end of the barbed suture was passed through the barrel of the needle into the nylon loop (Figure 2D), and the needle of a 16-gauge intravenous catheter was withdrawn at the end of the barbed thread and grasped using laparoscopic fundus grasping forceps (Figures 2E,F). Next, the nylon loop was removed from the abdomen using a barbed suture caught by the loop (Figures 3A,B). In this manner, both ends of the barbed suture exited through the skin puncture point (Figures 3C,D). The thread was then tied using a square knot (Figures 3E,F), buried under the skin. The same process was repeated three more times, resulting in a gastropexy suture length of 3 cm (Figures 3G,H). After deflation of the abdomen, the stay suture was removed, and the port sites were closed in a standard manner.

### 2.4 Surgical time

The TST was measured between the first skin incision and the end of port site closure. The GST was measured from immediately after the stay suture of the stomach to the last suture fulfillment.

### 2.5 Tensile tests

**Figure 4:**
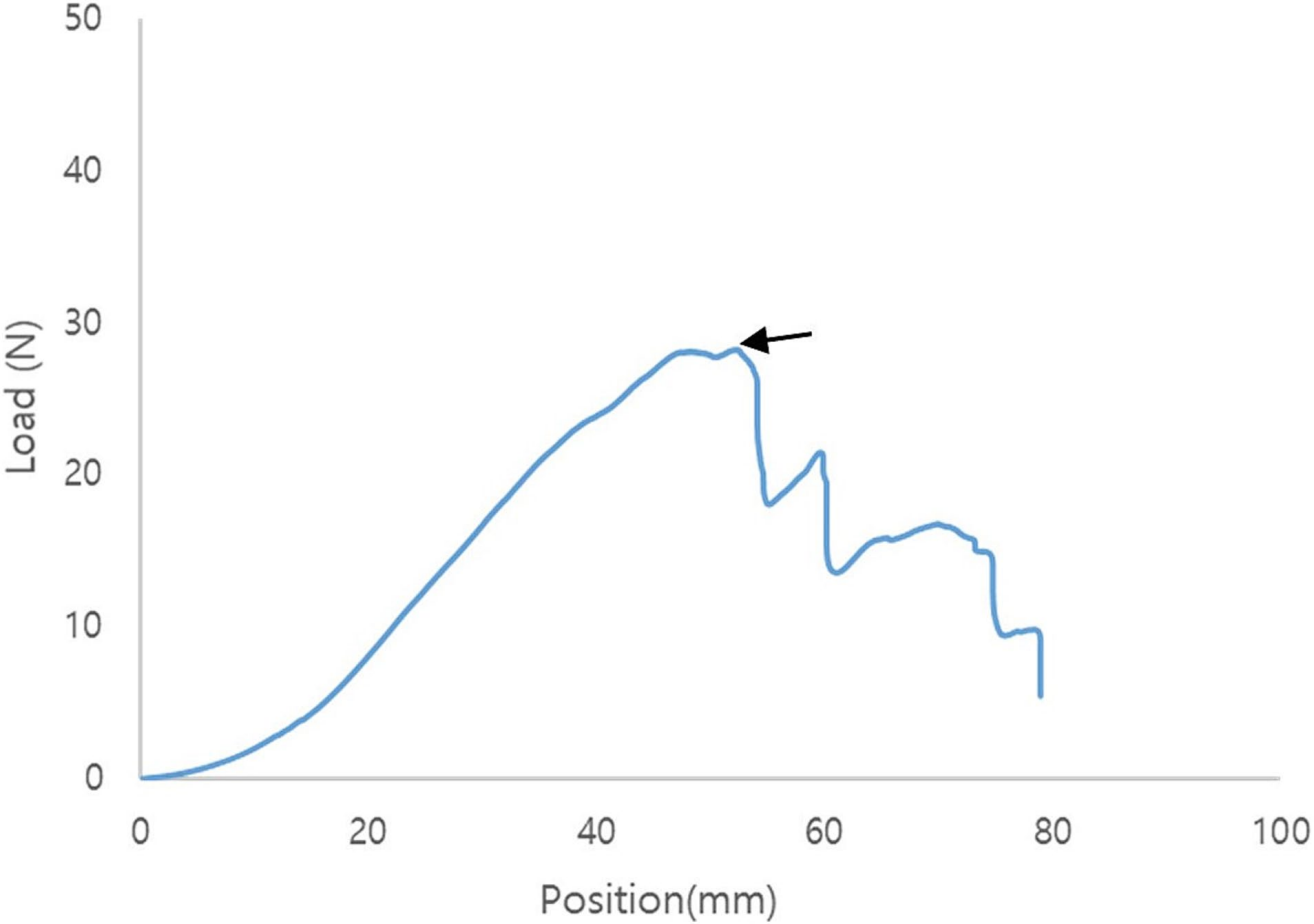
Representative tensile force curve of modified laparoscopic assisted percutaneous gastropexy (mLAPG). The arrow indicates the point of failure.

Immediately after surgery, the gastropexy area, which included the entire sutured area, abdominal wall, and gastric wall, was harvested. The abdominal wall comprises the transverse, internal, and external oblique muscles. The abdominal and gastric walls were excised using a 3 cm × 10 cm strip. These were linked together in the middle using sutures. During sampling, the intraluminal region was evaluated. The sample was immediately wrapped in lactated Ringer’s solution (LRS) soaked gauze sponge and stored at 5°C until the tensile force test. Within 6–12 h of collection, the samples were placed in a universal testing machine (AGS-X; Shimadzu Corp., Kyoto, Japan) with a pneumatic side-action grip to measure the tensile force. The strip-shaped samples were folded into a U-shape, wrapped, and sutured with gauze to increase the fractional force between the sample and grip. The samples were then grasped using the upper and bottom grips. The top grip was moved upward at a constant rate of 20 mm/min. Distraction was performed until maximum tension was reached. Tensile force during the test was recorded using Trapezium X software (Shimadzu, Kyoto, Japan). Failure was defined as the tearing of the tissue or suture and an untied knot. After the tests, the samples were examined to determine the occurrence of failure. At the point of failure, the load to failure was recorded in newtons (N) (Figure 4).

### 2.6 Statistical analysis

The Shapiro–Wilk test was used to analyze the data for normality. A two-group t-test power analysis was performed using the mean and standard deviation from the TLG and mLAPG groups to compare load-to-failure, total surgery time, and gastropexy suturing time. Statistical significance was set at *p* < 0.05.

## 3 Results

### 3.1 Suturing efficacy and outcomes

Suturing for gastropexy was successfully performed. A suture length of 3 cm and four bites were applied in both the TLG and mLAPG groups. No intraluminal sutures were found during harvesting of the abdominal wall and stomach. The knots of the mLAPG sutures were buried under the skin (Figure 3G).

### 3.2 Surgical time

For the mLAPG group, the TST was 61.83 ± 4.80 min (range: 55–71 min) and the GST was 31.33 ± 3.13 min (range: 27–38 min). For the TLG group, the TST was 65.33 ± 12.05 min (range: 50–85 min) and the GST was 37.5 ± 7.06 min (range: 28–48 min). The TST was not a significantly different between groups (*p* = 0.538), and the GST was also not significantly different between groups (*p* = 0.095).

### 3.3 Tensile strength tests

**Figure 5:**
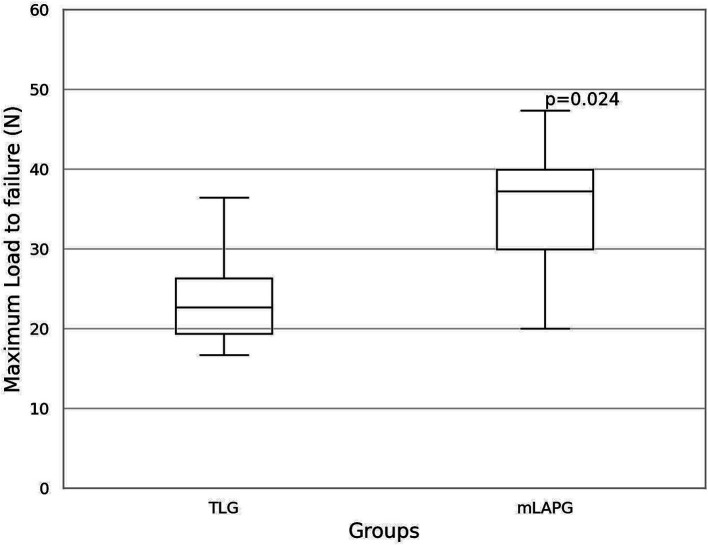
Box and whisker plot of the results of maximum load to failure (N) for total laparoscopic gastropexy (TLG) and modified laparoscopic assisted percutaneous gastropexy (mLAPG). The mLAPG group had a significantly higher maximum load to failure than the TLG group (*p* = 0.024).

The mean maximum load to failure required to disrupt adhesions was 24.04 ± 7.16 N for the TLG group and 35.86 ± 8.24 N for the mLAPG group (Figure 5). The mLAPG group had higher scores than the TLG group (*p* = 0.024). All failures occurred in the tissue owing to tearing of the seromuscular layer of the stomach at the pexy site. The sutures remained intact. No slippage occurred between the pneumatic side-action grips and samples.

## 4 Discussion

In this study, we modified laparoscopic-assisted percutaneous anterior gastropexy in infants (22) to be suitable for gastropexy in dogs and evaluated its feasibility in canine cadavers. Due to the difference in the volvulus type of the stomach, the pexy site has been determined to be a position known for prevent GDV in dogs, and a barbed suture, known to induce permanent adhesion at the pexy site, has been applied with an appropriate suture length and number. Our mLAPG did not involve intracorporeal suturing, making it technically less challenging than intracorporeal suturing of the TLG. This technique can be performed with a minimal two-port technique compared to the traditional TLG method and provides sufficient initial tensile force to induce adhesion at the gastropexy site.

To evaluate mLAPG, TLG was chosen as the control group because it is one of the popular minimally invasive methods. The choice of suture material, suture length, and number of suture bites in TLG was based on the most minimally invasive technique with reported successful outcomes. These terms were also set to be identical to those of mLAPG.

Several studies have examined the biomechanical characteristics and failure loads of various gastropexy techniques using either sacrificed live animals or cadavers (10, 14, 25–30). We used freshly thawed cadavers to evaluate biomechanical properties. Freezing and thawing of the tissues may have altered the tissue strength, and it is possible that the cadavers had already started to undergo autolysis before the procedures were performed. This likely affects the structural integrity of the stomach wall and causes it to tear at a lower force than anticipated in live dogs (26). Therefore, we measured the tensile force of the mLAPG and TLG in samples that were harvested and measured within 6–12 h after surgery in canine cadavers that underwent the same freezing and thawing processes.

Our distraction rate (20 mm/min) was consistent with that of a previous study (14, 31, 32). Samples for tensile tests were prepared and tested in a manner similar to that described by Mathon et al. (10). The maximum load to failure in the mLAPG group was higher than that in the TLG group, indicating successful clinical outcomes (8). This observation suggests that sufficient initial tension is provided at the pexy site of the mLAPG to induce successful adhesion.

Arbaugh et al. (25) demonstrated that the barbs of a knotless suture interact with the tissue to increase the load to the failure of gastropexy. The authors hypothesized that barbs in the suture likely increase the contact area between the suture and the tissue, which may reduce the pressure on the tissue. In the tensile test in this study, both mLAPG and TLG showed stomach tissue failure, and the mLAPG group had a higher load to failure than the TLG group, suggesting that the barbed suture had a wider contact area with the stomach tissue. In the TLG group, a 3/8 circle 24 mm suture needle was passed through the stomach, whereas in the mLAPG group, a straight 45 mm needle was passed through the stomach. This difference is presumed to result in a wider contact area between the barbed suture and the stomach in the mLAPG.

In a study of TLG using barbed sutures, failure occurred in the abdominal wall (25). However, in this study, no failures were observed in the abdominal walls. This suggests that our suturing technique that engages the barbed suture with the abdominal wall full-thickly may exert a stronger force between the barbed suture and abdominal wall.

To develop adhesions, tissue trauma is necessary for re-epithelialization (2). It was thought that tissue trauma in mLAPG occurred during the suturing process, and the interaction between the barbs of the sutures and the tissue resisting pull-out strength caused sufficient tissue trauma to induce adhesion. The extent of tissue damage is thought to increase proportionally with the length of the suture that interacts with the tissue. The length of the engaged suture with the same suture length and the same number of bites between the continuous pattern and our method was not significantly different. Furthermore, the absorbable knotless barbed suture (2-0 V-Loc 180) used in our experiment was confirmed to be suitable for inducing adhesion based on previous research (2, 3, 15). Therefore, if applied to live dogs, it is expected to provide sufficient trauma for adhesion under the same conditions of suture length and number of bites as applied a study by Giaconella et al. (8), who demonstrated intact total laparoscopic gastropexy.

The mLAPG has several additional advantages. TLG studies have demonstrated portal placement with conventional three-ports or SILS ports with one or two additional ports (2, 3, 5, 6, 8, 10, 11, 15). In some studies, relatively large ports were required to use special suturing devices (2, 5, 6). Our method requires only two 5 mm incisions for port placement. Therefore, our method allows for a minimal skin incision length, which could improve patient comfort by reducing surgical trauma and pain. In dogs, laparoscopy with two cannulas resulted in significantly less pain than that with three cannulas (34). To the best of our knowledge, this is the first laparoscopic gastropexy technique that uses two ports. Additionally, once the unidirectional barbed suture passes through the tissue, it engages with it, making it challenging to return it to its pre-passaged state. However, our suture technique has the advantage of modifying the position of the 18-gauge needle for the passage of the barbed suture.

Stay suture placement facilitated LAPG suturing by allowing visualization of the desired pexy site, eliminating the necessity of lifting the stomach using laparoscopic grasping forceps after stay suture placement, and preventing sutures from penetrating the stomach lumen. Despite the possibility of performing this method without stay sutures by manually elevating the stomach until the first LAPG knot is tied, this method was implemented owing to its advantages.

Considering the differences in surgical goals and the size of the subjects compared with the study performed in infants, we modified the suture material and the length and diameter of the needle used for mLAPG in dogs. Our mLAPG used a knotless barbed suture for greater tensile strength (25) and omitted additional incisions or abrasion processes (2, 8). Typically, a 3 or 3.5 metric size of suture materials are used for gastropexy (33). In a previous study, there was no statistically significant difference in the load to failure between 2 metric and 3 metric unidirectional barbed sutures in incisional gastropexy. However, Arbaugh et al. (25) reported that larger barbed sutures might provide a stronger load for failure. This is attributed to the increased contact area between the tissue and suture, which results in reduced pressure on the tissue. Therefore, instead of choosing a 2 metric unidirectional knotless barbed suture, a commonly used 3 metric unidirectional knotless barbed suture was selected. When 3 metric knotless unidirectional barbed sutures were passed through the lumen of an 18-gauge needle, there was significant resistance, whereas there was no resistance when passing through the lumen of a 16-gauge needle. Therefore, we decided to use a 16-gauge needle. We chose a commercially available 45 mm 16-gauge intravenous catheter needle for the mLAPG suturing process because it has sufficient length to penetrate the seromuscular layer of the stomach. However, considering that our experiment involved small dogs, it appears that for large or giant breeds, a longer needle length, possibly a spinal needle, may be necessary.

The mLAPG initially required less TST than the TLG. This may be because mLAPG does not require intracorporeal suturing, which requires advanced proficiency. The TST of both methods gradually decreased and reached a stable status over time; however, the mLAPG reached a stable status earlier than the TLG. mLAPG, although still showing some variability, seemed to have a relatively more consistent range of gastropexy suturing times. These findings suggest that the mLAPG procedure has a less challenging learning curve.

Our study has several limitations. First, the sample size of this study was relatively small. And second, due to the design of our study, it was not possible to evaluate the development of permanent adhesions between the stomach and body wall and the progression of complications. Further evaluation in clinical cases is required to confirm adhesion formation by laparoscopy or ultrasonography and the occurrence of complications.

## 5 Conclusion

Our mLAPG technique was performed without the intracorporeal knot-tying process using two ports and had superior loads to failure compared to TLG. This finding suggests the potential use of mLAPG for minimally invasive prophylactic gastropexy in live dogs. However, further evaluation of clinical cases is required.

## Data Availability

The raw data supporting the conclusions of this article will be made available by the authors, without undue reservation.
